# Does Unilateral Oocyte Retrieval due to Transvaginally Inaccessible Ovaries, Contrary to Common Beliefs, Affect IVF/ICSI Treatment Outcomes That Much?

**DOI:** 10.1155/2016/3687483

**Published:** 2016-03-30

**Authors:** Safak Olgan, Sezcan Mumusoglu, Gurkan Bozdag

**Affiliations:** ^1^IVF Unit, Department of Obstetrics and Gynecology, School of Medicine, Akdeniz University, 07059 Antalya, Turkey; ^2^IVF Unit, Department of Obstetrics and Gynecology, School of Medicine, Hacettepe University, 06100 Ankara, Turkey

## Abstract

*Objective*. To investigate in vitro fertilization (IVF) treatment outcomes of unilateral oocyte retrieval in patients with transvaginally inaccessible ovaries.* Study Design*. Ninety-two women who underwent unilateral oocyte retrieval were retrospectively matched for age, antral follicle count, and body mass index with 184 women who underwent bilateral oocyte retrieval. Each patient in bilateral oocyte retrieval group had the same number of cumulus oophorus complexes (COCs) from single ovary and had comparable number of follicles (±2) on contralateral site where follicular aspiration was performed.* Results*. The number of COCs, metaphase-2 oocytes, 2-pronuclei, and top-quality embryos was significantly lower in unilateral oocyte retrieval group. However, proportion of patients with an embryo transfer of at least one top-quality embryo was found to be comparable between unilateral and bilateral oocyte retrieval. Subsequently, clinical pregnancy and live birth rates were found to be similar between the groups. The ROC curve analysis revealed (AUC = 0.74, 95% CI 0.63–0.86, *p* = 0.001) that retrieved COCs ≥ 5 from single ovary had sensitivity of 76.0% and specificity of 64.2% for occurrence of a clinical pregnancy.* Conclusion*. The patients with unilateral oocyte retrieval have reasonable chance of success with IVF. The retrieval of ≥5 COCs from accessible ovary might result in better treatment outcomes among these patients.

## 1. Introduction

Ovarian stimulation is an important part of assisted reproduction treatment (ART). This regimen is used to stimulate multiple follicle development in a single cycle [1]. Increasing the number of mature oocytes suitable for IVF procedures improves the likelihood of generating good quality embryos and of achieving a successful pregnancy [1, 2]. Previous studies suggested that between 8 and 15 retrieved oocytes may be the optimal number in a fresh IVF cycle in order to maximize treatment success and minimize the cycle cancellation rate [3, 4].

Transvaginal oocyte retrieval, under ultrasound guidance, is the current method of choice for oocyte retrieval in IVF and intracytoplasmic sperm injection (ICSI) [5]. It is generally a safe and a well-tolerated procedure associated with a low overall complication rate, commonly cited at less than 5%. The most frequent complications are vaginal bleeding, mild pelvic infection, and pelvic abscess. The less common, but more serious, complications include intra-abdominal hemorrhage and trauma to pelvic structures including the bowel, bladder, and large vessels [5–12]. However, there is the possibility of the underreporting of complications, and this may be attributed to the rare nature of this event and to the fact that many IVF treatment providers may be less interested in reporting complications because the major scientific focus is on IVF-ICSI outcomes.

Transvaginal oocyte retrieval, however, poses a bothersome problem when the ovaries are not accessible transvaginally due to variations in pelvic organ anatomy from biological variability or pelvic/abdominal disease. As the clinicians attempt to obtain the maximum number of oocytes in line with their patients' reproductive potential, those patients with inaccessible single ovaries might be more prone to complications related to oocyte pick-up procedures. However, the issue is whether it is possible to improve the IVF treatment outcomes in each and every patient by increasing the available oocytes. Since the nomograms for the optimum number of oocytes were generated considering the general infertile population, including poor responders, treatment outcomes for unilateral oocyte retrieval cases might not reflect their true IVF success. Unilateral oocyte retrieval might still have a higher reproductive potential than the corresponding bilateral oocyte retrieval with the same number of oocytes. Moreover, increasing the number of eggs from the inaccessible ovary might not improve IVF outcomes, at least in a subgroup of patients with an adequate number of eggs retrieved from the safely accessible side. This study therefore aims to investigate the treatment outcomes of single versus bilateral oocyte retrieval and define a minimum number of oocytes from a single ovary for which further procedures might be abandoned due to safety concerns.

## 2. Materials and Methods

This retrospective study included patients who underwent IVF treatment at the Hacettepe University IVF Centre between January 2010 and December 2014. The electronic database was screened to identify all the women with unilateral oocyte retrieval during the study period. The procedure was not performed in one ovary, as its position was far from the transvaginal ultrasound probe, and it was therefore judged by the operator as dangerous for the patient. Ninety-two ART cycles with unilateral oocyte retrieval were identified. In order to reflect routine clinical practice, no exclusion criteria were imposed on this dataset. Each patient in the unilateral oocyte retrieval group was individually matched with two bilateral oocyte retrieval patients during the same period. The control subjects were two women, closest in time to the index case who were matched for age (±2), antral follicle count (≤5, 6–15, ≥16), and body mass index (BMI), according to World Health Organization (WHO) categories. Each patient in the bilateral oocyte retrieval group had the same number of cumulus oophorus complexes (COCs) from a single ovary and had a comparable number of ≥14 mm follicles (±2) on the contralateral site where follicular aspiration was performed. The researchers who identified the patients for inclusion and matched study subjects with controls were blinded to the oocyte yield and pregnancy status of the patients.

While this research was submitted for institutional review board (IRB) approval, it was exempted because of its retrospective, noninterventional nature: no patients were contacted, and no identifying patient information was used for purposes of this study. Patients underwent IVF according to standard stimulation protocols, which involved pituitary downregulation with a gonadotropin-releasing hormone (GnRH) agonist (Lucrin; Abbott Cedex, Istanbul, Turkey) administered in the mid-luteal phase of the prior cycle (long protocol) or a diluted GnRH agonist on days 2–4 of the cycle (microdose protocol). Alternatively, the GnRH antagonist (Cetrotide; Merck, Istanbul, Turkey, or Orgalutran; MSD, Istanbul, Turkey) short protocols started on the 5th day of stimulation. Controlled ovarian stimulation was achieved with a recombinant follicle-stimulating hormone (FSH) (Gonal-F; Serono, Istanbul, Turkey, or Puregon; MSD, Istanbul, Turkey) and/or a urinary FSH (Menogon, Ferring, Istanbul, Turkey). The starting dose of gonadotropin was determined based on the age of the female, antral follicle count at baseline transvaginal ultrasonography, day 3 FSH and estradiol (E2) levels, BMI, and previous ovarian response, if available. The response to stimulation was monitored using serum E2 and transvaginal ultrasound. The criterion for human chorionic gonadotropin (hCG) (Pregnyl; MSD, Istanbul, Turkey, or Ovitrelle; Merck Serono, Istanbul, Turkey) administration was the presence of three or more follicles exceeding 17 mm in diameter for both groups. Oocyte retrieval was carried out under local anaesthesia using vaginal ultrasound-guided puncture of follicles 36 h after hCG administration. Standard procedures were carried out for gamete-embryo handling. The embryos were transferred to the uterus either 3 days (cleavage stage) or 5 days (blastocyst stage) after the oocyte retrieval using a soft catheter (Wallace, PM Group, Istanbul, Turkey), and the embryo quality was assessed on the day of the transfer. The cleavage stage embryos were graded according to their cell number and the degree of fragmentation [14]. The embryos at an appropriate developmental stage, with <20% fragments and a mild degree of uneven-sized blastomeres (grade I, grade IIA, and grade IIB), constituted the high quality cleavage stage embryos. In the case of extended cultures, all of the blastocyst stage embryos were evaluated using the grading system of Gardner and Schoolcraft (1999). The blastocysts were graded according to the degree of expansion and the quality of the inner cell mass and trophectoderm [15]. The grade III–VI embryos, according to blastocyst expansion and hatching status, with an inner cell mass and trophectoderm grades of A or B, constituted the high quality blastocysts. The luteal phase was supported by daily vaginal progesterone suppositories (Crinone; Merck Serono, Istanbul, Turkey) starting 1 day after oocyte pick-up.

Clinical pregnancy was defined as the presence of an intrauterine sac with fetal heart activity at 6–8 weeks of gestation. Live birth was defined as the delivery of a live infant after 24 weeks of gestation. Miscarriage rate was defined as a pregnancy loss before 20 weeks of gestation per patient with a positive *β*-hCG test (≥25 IU/L).

The data were analyzed using the SPSS for Windows version 22.0. The distribution of continuous variables is described with the use of the mean and the standard deviation (SD). Categorical variables are presented as percentages of the total. Comparison of continuous variables among groups was performed with the use of the unpaired Student's *t*-test or the Mann-Whitney *U* test depending on the normality of the distribution, while Pearson's chi-square test or Fisher's exact test was used to compare categorical variables. Receiver operator characteristic (ROC) curve analysis was used to assess the predictive value of retrieved eggs in the unilateral oocyte aspiration group and the occurrence of clinical pregnancy. The percentage of area under curve (AUC) and confidence intervals (CI 95%) were generated for the ROC curve. Discrimination threshold was chosen on the basis of optimal sensitivity and specificity. A *p* value <0.05 was considered statistically significant.

## 3. Results

Table 1 describes the demographical and fertility characteristics between the unilateral and bilateral oocyte retrieval groups. There was no significant difference in age, BMI, antral follicle count, duration of infertility, prior pregnancy, number of previous unsuccessful attempts at IVF, polycystic ovaries at ultrasound, presence of endometriosis, and poor ovarian reserve between the groups (*p* > 0.05 for each).

IVF-ICSI cycle characteristics revealed that the number of COCs (*p* < 0.001), metaphase 2 (MII) oocytes (*p* < 0.001), 2-pronuclei (2PN) (*p* < 0.001), and top-quality embryos (*p* < 0.001) was significantly higher in the bilateral oocyte retrieval group (Table 2). The unilateral oocyte retrieval group had significantly higher cycle cancellation rates due to the lack of MII oocytes, 2PN, or embryos suitable for transfer (*p* = 0.050). Accordingly, the number of patients who reached embryo transfer was lower in the unilateral oocyte retrieval group (*p* = 0.045). The mean number of embryos transferred was found to be similar among the groups (*p* > 0.05). The bilateral oocyte retrieval group had a significantly increased number of transferred embryos at the blastocyst stage (*p* = 0.001). However, the proportions of patients with at least one top-quality embryo, in either the cleavage or blastocyst stages, were comparable between unilateral and bilateral oocyte retrieval groups (*p* > 0.05) (Table 2). Clinical pregnancy and live birth rates were similar between the groups (*p* > 0.05 for each). Pregnancy outcomes, both per patient reaching oocyte retrieval and per embryo transfer (ET), are shown in Table 3.

**Figure 1:**
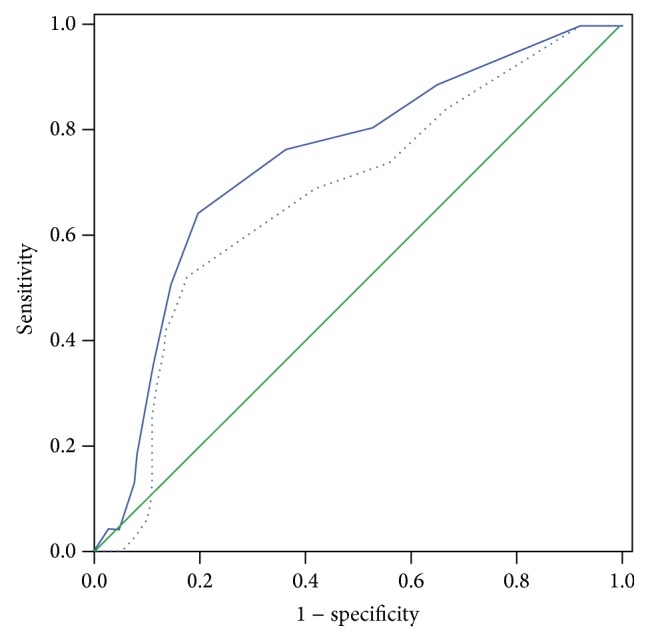
Receiver operator characteristic curves for cumulus oophorus complexes to predict clinical pregnancy (straight line) and live birth (dotted line).

ROC analysis showed a significant relationship between the number of COCs and the occurrence of a clinical pregnancy (AUC = 0.74, CI 95% 0.63–0.86, *p* < 0.001) and a live birth (AUC = 0.67, CI 95% 0.54–0.81, *p* = 0.020) (Figure 1). When COCs ≥ 5 IU/L, sensitivities were 76.0% versus 68.4%, specificities were 64.2% versus 58.9%, positive predictive values (PPV) were 44.2% versus 30.2%, and negative predictive values (NPV) were 87.8% versus 87.8%, for clinical pregnancies and live births, respectively.

## 4. Discussion

The bilateral oocyte retrieval group had a significantly higher number of MII oocytes, 2PN, and cleavage stage embryos, as well as decreased cycle cancellation rates due to the lack of embryos suitable for transfer. However, these findings do not indicate higher pregnancy rates when compared to unilateral oocyte retrieval. This might be due to the concurrent increase in medium-low grade embryos which results in the discarding of a significant proportion of embryos due to their unsuitability for transfer. Additionally, the proportion of the patients' ET of at least one top-quality embryo was comparable between the groups. Several studies have indicated that the quality and quantity of embryos are the two most important predictors of fresh IVF-ICSI cycles [16, 17]. As previously suggested, this might imply that the number and quality of embryos transferred might reveal the effect that the number of oocytes retrieved has on pregnancy after fresh ET [18]. Consequently, the chances of achieving a clinical pregnancy and live birth after fresh ET are independent of the number of oocytes retrieved if the patients have the same number and quality of embryos transferred [18]. Finally, it was proposed that the relationship between the number of oocytes and pregnancy in a fresh IVF cycle assumed an increasing then plateauing pattern [1, 18]. Although the underlying functional form of the relationship between the numbers of oocytes and IVF outcomes is nonlinear in the general population [4, 18], the relationship needs to be redetermined in unilateral oocyte retrieval patients. When ROC curves were generated in the bilateral oocyte retrieval group for COCs in order to discriminate between a clinical pregnancy and a live birth, AUC values were found to be 53.2 and 53.0, respectively (data are not presented). Low AUC values indicated that the ability of these models to discriminate was limited and implied low model accuracy. However, by using the ROC curve analysis in the unilateral group, we found that the optimal number of oocytes should be ≥5 (AUC values were 0.74 and 0.67, resp.) in order to increase clinical pregnancy and live birth rates.

It should be noted that transvaginal inaccessibility of the ovaries is uncommon; indeed, this occurred in less than 0.4–1.6% of oocyte retrievals [19, 20]. This study reviewed more than 2,000 IVF cycles over a period of five years and identified 92 cases with unilateral oocyte retrieval. The higher prevalence of inaccessible ovaries in this study might be due to the inclusion of cases with a single follicle in the inaccessible site (28 patients). Therefore, the negligible number of follicles in the inaccessible ovary might create a clinical scenario that has been previously ignored by IVF practitioners. An alternative method that has been developed for these cases is transabdominal ultrasound-guided retrieval [19, 20]. Despite the feasibility of this approach, several drawbacks still exist including the need for additional skills as more than one operator was required to complete the process (one to use the ultrasound and a second to aspirate the follicles). Additionally, multiple transabdominal wall punctures still potentially increase the risk of bowel and vessel injury [20].

By matching cases and controls for age, BMI, and antral follicle count, we minimized any differences accounted for by characteristics other than the unilateral versus bilateral retrieval. However, IVF outcomes are influenced by a variety of patient characteristics, and this study was unable to control for all of these potential confounders including patient history of previous ovarian surgery. Subsequently, the unilateral oocyte retrieval group consists of patients with a worse prognosis in this study, but this limitation does not affect the major conclusion. Also, this study only analyzed the outcome of fresh IVF cycles, and it did not take into account the impact of frozen-thawed cycles on the cumulative live birth rate. The proportions of patients with excess embryos for freezing were found to be comparable between the groups.

## 5. Conclusion

It is reassuring that pregnancy rates were not significantly different between controls and cases; however, this information is provided primarily to illustrate that these patients have a reasonable chance of success with IVF, not to promote unilateral oocyte retrieval. Moreover, retrieval of ≥5 COCs from a single ovary might be encouraging for better IVF treatment outcomes among these patients. Thus, the decision to abandon follicular aspiration from the single inaccessible ovary or use alternative methods should depend on the number of retrieved COCs, the residual follicles in the inaccessible ovary, and the anticipation of patient risk factors during any further procedures.

## Figures and Tables

**Table 1:** Demographics and fertility characteristics of patients in unilateral versus bilateral aspiration groups.

	Unilateral aspiration (*n* = 92)	Bilateral aspiration (*n* = 184)	*p*
Age (years)	33.2 ± 5.0	33.2 ± 5.2	0.980
BMI (kg/m^2^)	27.2 ± 4.4	27.1 ± 4.1	0.733
Previous unsuccessful attempts at IVF	1.4 ± 0.8	1.4 ± 0.7	0.952
Antral follicle count	14.9 ± 9.1	14.7 ± 8.3	0.854
Ever pregnant prior to inclusion	24 (26.1)	63 (34.2)	0.169
Duration of infertility (years)	5.1 ± 4.0	5.8 ± 4.5	0.162
Baseline FSH (IU/L)	6.3 ± 2.7	6.6 ± 4.1	0.184
Baseline LH (IU/L)	5.7 ± 3.7	5.3 ± 3.1	0.790
Baseline estradiol (pg/mL)	62.6 ± 33.0	59.8 ± 30.2	0.232
Endometriosis^a^	10 (10.9)	9 (4.9)	0.064
Previous ovarian surgery	10 (10.9)	6 (3.3)	**0.011**
Polycystic ovaries on ultrasound	24 (26.1)	50 (27.2)	0.848
Poor ovarian reserve^b^	9 (9.8)	15 (8.2%)	0.650

BMI, body mass index; IVF, in vitro fertilization. Data are presented as mean ± standard deviation or *n* (%). Boldface data indicates statistical significance (*p* < 0.05).

^a^Laparoscopically confirmed endometriosis or presence of an endometrioma.

^b^According to Bologna criteria (2011) [13].

**Table 2:** IVF/ICSI cycle characteristics between unilateral and bilateral oocyte retrieval.

	Unilateral aspiration (*n* = 92)	Bilateral aspiration (*n* = 184)	*p*
Duration of stimulation (days)	9.7 ± 2.1	9.6 ± 2.0	0.447
Gonadotropin dose (IU)	2595.0 ± 802.5	2497.5 ± 885.0	0.354
Total follicles on day of hCG administration	11.7 ± 7.7	11.4 ± 7.5	0.480
COCs	4.9 ± 3.2	9.7 ± 5.6	**<0.001**
MII oocytes (MII)	4.3 ± 2.8	8.1 ± 4.7	**<0.001**
Maturation rate (MII/COCs) % per patient	87.1 ± 18.6	84.7 ± 19.5	0.302
2-pronuclei (2PN) oocytes	3.4 ± 2.4	6.2 ± 3.8	**<0.001**
Fertilization rate (2PN/MII) % per patient	78.1 ± 27.8	76.0 ± 18.6	0.496
Embryo quality on days 2-3 of in vitro culture			
Top-quality embryos	2.5 ± 1.9	4.4 ± 3.5	**<0.001**
Medium-low quality embryos	0.9 ± 1.4	1.9 ± 2.4	**<0.001**
Cancelled cycles			
No MII oocytes, 2PN, or embryos	8 (8.7)	6 (3.3)	**0.050**
Risk of OHSS (freeze all)	1 (1.1)	1 (0.5)	1.000
Patients with embryo transfer	83 (90.2)	177 (96.2)	**0.045**
Number of embryos transferred	1.3 ± 0.5	1.3 ± 0.5	0.648
Day of embryo transfer			
Days 2-3	71 (85.5)	118 (66.7)	**0.001**
Day 5	12 (14.5)	59 (33.3)	
Proportion of patients with ET of at least one top-quality cleavage stage embryo	56 (78.9)	96 (81.4)	0.677
Proportion of patients with ET of at least one top-quality blastocyst	9 (75.0)	38 (64.4)	0.739
Proportion of patients with excess embryos for freezing	10 (10.9)	31 (16.8)	0.188

Data are presented as mean ± standard deviation or *n* (%). hCG, human chorionic gonadotropin; COCs, cumulus oocyte complexes; MII, metaphase 2; 2PN, 2-pronuclei; OHSS, ovarian hyperstimulation syndrome; ET, embryo transfer; ICSI, intracytoplasmic sperm injection. Boldface data indicates statistical significance (*p* < 0.05).

**Table 3:** Pregnancy outcomes between unilateral and bilateral oocyte retrieval.

Pregnancy outcomes	Unilateral aspiration (*n* = 92)	Bilateral aspiration (*n* = 184)	*p*
Positive hCG per ET	30 (36.1)	70 (39.5)	0.599
Positive hCG per patient reaching oocyte retrieval	30 (32.6)	70 (38.0)	0.376
Clinical pregnancy per ET	25 (30.1)	58 (32.8)	0.669
Clinical pregnancy per patient reaching oocyte retrieval	25 (27.2)	58 (31.5)	0.458
Live birth per ET	19 (22.9)	47 (26.6)	0.527
Live birth per patient reaching oocyte retrieval	19 (20.7)	47 (25.5)	0.369
Miscarriage rate per positive hCG	11 (36.7)	23 (32.9)	0.712

Data are presented as *n* (%). ET, embryo transfer; hCG, human chorionic gonadotrophin.

## References

[1] Macklon N. S., Stouffer R. L., Giudice L. C., Fauser B. C. J. M. (2006). The science behind 25 years of ovarian stimulation for in vitro fertilization. *Endocrine Reviews*.

[2] Fauser B. C. J. M., Devroey P., Macklon N. S. (2005). Multiple birth resulting from ovarian stimulation for subfertility treatment. *The Lancet*.

[3] Ji J., Liu Y., Tong X. H., Luo L., Ma J., Chen Z. (2013). The optimum number of oocytes in IVF treatment: an analysis of 2455 cycles in China. *Human Reproduction*.

[4] Sunkara S. K., Rittenberg V., Raine-Fenning N., Bhattacharya S., Zamora J., Coomarasamy A. (2011). Association between the number of eggs and live birth in IVF treatment: an analysis of 400 135 treatment cycles. *Human Reproduction*.

[5] Siristatidis C., Chrelias C., Alexiou A., Kassanos D. (2013). Clinical complications after transvaginal oocyte retrieval: a retrospective analysis. *Journal of Obstetrics and Gynaecology*.

[6] Catanzarite T., Bernardi L. A., Confino E., Kenton K. (2015). Ureteral trauma during transvaginal ultrasound-guided oocyte retrieval: a case report. *Female Pelvic Medicine and Reconstructive Surgery*.

[7] Nouri K., Walch K., Promberger R., Kurz C., Tempfer C. B., Ott J. (2014). Severe haematoperitoneum caused by ovarian bleeding after transvaginal oocyte retrieval: a retrospective analysis and systematic literature review. *Reproductive BioMedicine Online*.

[8] Patounakis G., Krauss K., Nicholas S. S., Baxter J. K., Rosenblum N. G., Berghella V. (2012). Development of pelvic abscess during pregnancy following transvaginal oocyte retrieval and in vitro fertilization. *European Journal of Obstetrics Gynecology and Reproductive Biology*.

[9] Jayakrishnan K., Raman V. K., Vijayalakshmi V. K., Baheti S., Nambiar D. (2011). Massive hematuria with hemodynamic instability—complication of oocyte retrieval. *Fertility and Sterility*.

[10] Aragona C., Mohamed M. A., Espinola M. S. B. (2011). Clinical complications after transvaginal oocyte retrieval in 7,098 IVF cycles. *Fertility and Sterility*.

[11] Bozdag G., Basaran A., Cil B., Esinler I., Yarali H. (2008). An oocyte pick-up procedure complicated with pseudoaneurysm of the internal iliac artery. *Fertility and Sterility*.

[12] Kelada E., Ghani R. (2007). Bilateral ovarian abscesses following transvaginal oocyte retrieval for IVF: a case report and review of literature. *Journal of Assisted Reproduction and Genetics*.

[13] Ferraretti A. P., La Marca A., Fauser B. C. J. M., Tarlatzis B., Nargund G., Gianaroli L. (2011). ESHRE consensus on the definition of ‘poor response’ to ovarian stimulation for in vitro fertilization: the Bologna criteria. *Human Reproduction*.

[14] Hardarson T., Hanson C., Sjögren A., Lundin K. (2001). Human embryos with unevenly sized blastomeres have lower pregnancy and implantation rates: indications for aneuploidy and multinucleation. *Human Reproduction*.

[15] Gardner D. K., Schoolcraft W. B. (1999). In vitro culture of human blastocyst. *Towards Reproductive Certainty: Infertility and Genetics Beyond*.

[16] Cai Q. F., Wan F., Huang R., Zhang H. W. (2011). Factors predicting the cumulative outcome of IVF/ICSI treatment: a multivariable analysis of 2450 patients. *Human Reproduction*.

[17] Cai Q., Wan F., Appleby D., Hu L., Zhang H. (2014). Quality of embryos transferred and progesterone levels are the most important predictors of live birth after fresh embryo transfer: a retrospective cohort study. *Journal of Assisted Reproduction and Genetics*.

[18] Cai Q., Wan F., Huang K., Zhang H. (2013). Does the number of oocytes retrieved influence pregnancy after fresh embryo transfer?. *PLoS ONE*.

[19] Barton S. E., Politch J. A., Benson C. B., Ginsburg E. S., Gargiulo A. R. (2011). Transabdominal follicular aspiration for oocyte retrieval in patients with ovaries inaccessible by transvaginal ultrasound. *Fertility and Sterility*.

[20] Edris F., Holiva N., Baghdadi S. (2014). Single operator ultrasound guided transabdominal oocyte retrieval in patients with ovaries inaccessible transvaginally: a modified technique. *Gynecology & Obstetrics*.

